# Salvage transoral laser microsurgery for early local recurrence of glottic squamous cell cancer

**DOI:** 10.1186/s40463-023-00628-7

**Published:** 2023-05-29

**Authors:** Zhimou Cai, Huijun Yue, Lin Chen, Yang Xv, Yun Li, Bingjie Tang, Yu Lin, Wenbin Lei

**Affiliations:** Department of Otolaryngology, The First Affiliated Hospital of Sun Yat-Sen University, No.58 Zhongshan Er Road, Guangzhou, 510080 Guangdong People’s Republic of China

**Keywords:** Glottic cancer, Transoral laser microsurgery, Open partial laryngectomy, Local recurrence

## Abstract

**Background:**

For recurrent laryngeal cancer, the feasibility of salvage transoral laser microsurgery (TLM) remains controversial. This study compared the efficacy of TLM and open partial laryngectomy (OPL) for treatment of early local recurrence of glottic squamous cell cancer (GSCC) and confirm the effectiveness of salvage TLM as a treatment option.

**Methods:**

This retrospective study involved 55 patients with early local recurrent GSCC treated with TLM, and the oncologic outcomes, functional outcomes, hospitalization time and complications were compared with a group of 40 recurrent GSCC patients matched for clinical variables of TLM group, treated by OPL by the same team of surgeons.

**Results:**

The 5-year overall survival and disease-specific survival rates were 65.8% and 91.5%, respectively, for 55 patients with rT_is_–rT_2_ stage treated by TLM and 77.1% and 94.7%, respectively, for 40 patients with rT_is_–rT_2_ stage treated by OPL (OPL group). In the TLM and OPL groups, the local control rates after 5 years were 77.5% and 79.3%, respectively, and the laryngeal preservation rates were 94.4% and 83.6%, respectively (*p* > 0.05). Compared with the OPL group, the complication rate (1.82%) and hospitalization duration (5.42 ± 2.26 days) were significantly lower in the TLM group (*p* < 0.05). Compared with the OPL group, postsurgical health-related quality of life and quality of voice were significantly better in the TLM group (*p* < 0.001).

**Conclusion:**

Salvage TLM can be used as an effective treatment option for suitable patients after a full, comprehensive, and careful assessment of the characteristics of early locally recurrent glottic carcinoma.

**Graphical Abstract:**

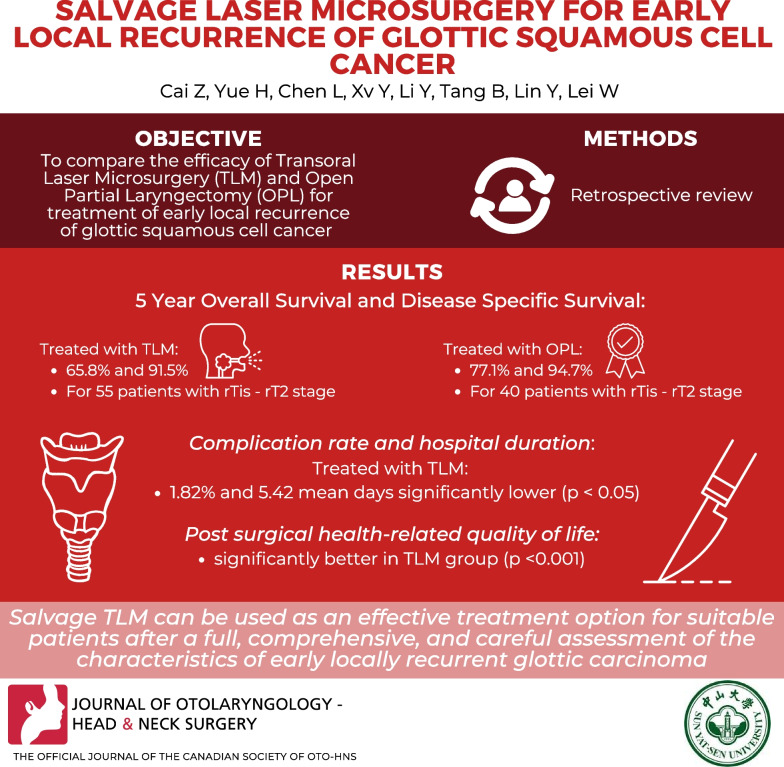

**Supplementary Information:**

The online version contains supplementary material available at 10.1186/s40463-023-00628-7.

## Background

The recurrence rate for laryngeal cancer following successful initial treatment is as high as 10–50%, and salvage surgery is preferred for comprehensive treatment [1–3]. Recurrent laryngeal cancer is considered dangerous and life-threatening. Hence, total laryngectomy as a salvage surgery has been recommended, which necessitates a tracheal stoma that can lead to permanent aphonia, resulting in a poor quality of life (QOL) [4–6]. Opinions regarding treatment modalities have changed following the discovery that for some recurrent laryngeal cancers, especially early-stage cancer, open partial laryngectomy (OPL) is feasible and enables laryngeal function preservation [7, 8]. However, the incidence of postoperative complications in OPL is also high; some patients develop laryngeal stenosis requiring a permanent tracheotomy [9, 10].

In transoral laser microsurgery (TLM), the surgical site is accessed directly through the natural human body cavity, greatly reducing the patient’s postoperative pain and surgical complication incidence. It can better preserve vocalization and swallowing functions under the premise of complete removal of the laryngeal tumor and maintenance of a high local control rate (LCR), which greatly improves the post-surgical QOL of the patients. Currently, radical radiotherapy or TLM are recognized as the first choice for early glottic cancer treatment owing to its effectiveness and minimally damage advantages [11–14]. However, for recurrent laryngeal cancer, the feasibility of salvage TLM remains controversial.

Some scholars have pointed out that TLM may be an effective treatment for early recurrent glottic cancer to preserve laryngeal function [15–17]. However, the number of cases reported in the literature for salvage treatment using TLM remains small, and there has been no comparative analysis between TLM and conventional open surgery. Additionally, few reports exist on the assessment of the patients’ voice and QOL after TLM salvage treatment.

Therefore, we aimed to evaluate the oncology results of patients with early local recurrence of glottic squamous cell cancer (GSCC) who received TLM or OPL salvage treatment at a single institution to determine: (1) whether TLM is feasible for early local recurrence of GSCC, (2) the differences between the oncology results after TLM and OPL treatment, (3) and whether patients have a better quality of voice (QOV) and QOL after undergoing TLM compared to OPL.

## Patients and methods

### Study design, inclusion, and exclusion of patients

This study was approved by the the Sun Yat-sen University Ethics Committee for Research and Publication. As all data were anonymized and retrospectively collected, informed consent of the participants was not required.

The medical records of all patients with early local recurrence (rT_is_-rT_2_) of GSCC who underwent TLM or OPL salvage treatment between January 2013 and January 2019 at the Department of Otorhinolaryngology at our hospital were analyzed retrospectively.

Patients included were those: (1) with early local recurrence of GSCC and pathological biopsy results confirming the pathological results of the first treatment, (2) assessed as rT_is_N_0_M_0_ – rT_2_N_0_M_0_ according to the American Joint Committee on Cancer staging [18]. Exclusion criteria were patients with: (1) non-squamous cell carcinoma or other malignant tumors, (2) distant metastases, or (3) inoperable tumors.

### Operative technique and follow-up

**Fig. 1:**
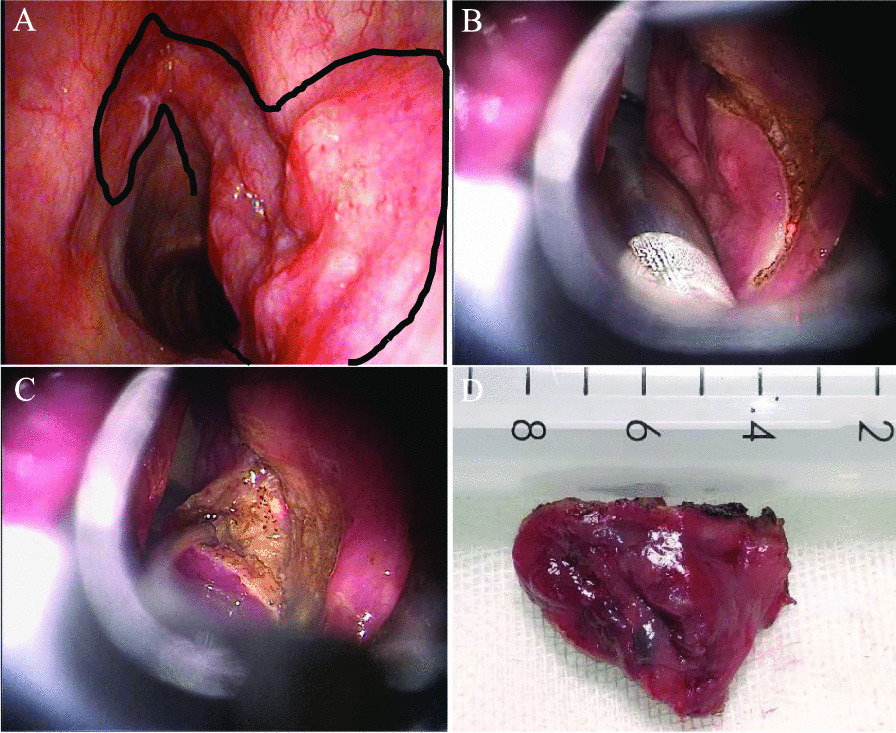
Salvage transoral laser microsurgery for early local recurrent glottic squamous cell cancer **A, B** Accurately delineating the boundary of the lesion with the CO_2_ laser. **C**, **D** Complete removal of the tumor

The surgical process was as follows. Electronic laryngoscopy, enhanced computed tomography, and magnetic resonance imaging were used to evaluate the extent and depth of the patients’ tumors and exclude thyroid cartilage infiltration or extension to the paraglottic and pre-epiglottic spaces. During the operation, the different features of the self-retaining laryngoscope were replaced based on individual need to permit full tumor exposure. A multi-angle (0°, 30°) endoscope with narrow-band imaging (NBI) was used to assist in the evaluation of the range of lesion resection. In the CO_2_ laser single-shot mode, the resection range of the lesion was marked, and the safety margin was set at 8–10 mm. The imaging data were combined to determine the depth of resection at the base of the lesion. If the lesion was not deeply infiltrated, the distance of the resection margin was set at 3–5 mm. The excision was carried out using a small-spot (0.3 mm) laser on repeated-pulse mode, and a bloodless surgery was performed to ensure the complete resection of the tumor. Following the total resection of the lesion, the surgical margins were marked at multiple points, and a frozen section of the margin specimen was obtained to ensure clean removal of the tumor (Fig. 1, Additional files 1, 2: Video P1, P2).

The patients in the OPL group were treated with supracricoid partial laryngectomy (13/23) and vertical partial laryngectomy (10/23), which removed the laryngeal tumor while retaining the laryngeal pronunciation, respiration, and swallowing functions.

All patients underwent regular otorhinolaryngology outpatient follow-up. Following the salvage surgery, monthly follow-up was conducted for the first 6 months, followed by every 3 months for the next 18 months, and then semiannually for the next 36 months.

### Questionnaires

The postsurgical health-related QOL (HRQOL) and QOV of the patients were evaluated by the European Organization for Research and Treatment of Cancer Quality of Life Questionnaire-Head and Neck (EORTC QLQ-H&N35) scales and the Voice Handicap Index (VHI), respectively, at least 12 months postoperatively. The Chinese version of the EORTC QLQ-HN&35 has been tested for reliability and validity in Chinese patients with cancer and can be used to evaluate the HRQOL of Chinese patients [19]. According to the EORTC QLQ-C30 grading manual (Version 3), the primary score of the scale should be converted to a centesimal system, where higher scores are related to worse HRQOL [20, 21].

VHI is recognized as the gold standard for QOV evaluation of patients with voice disorders [22]. The higher the score, the less satisfied the patient is with their voice [23]. This study used the Chinese version of the VHI-30 scale, and the credibility of the scale has been verified in previous studies [24].

### Statistical analysis

All statistical analyses were processed using IBM SPSS version 21 software (IBM, Armonk, NY).

Since the scale score distribution was non-normal (Kolmogorov–Smirnov test, *p* < 0.05), the Mann − Whitney U test was used to analyze the differences in HRQOL and QOV between the different groups of patients.

Time-to-event analyses were calculated using a Kaplan − Meier method to estimate the overall survival (OS), disease-specific survival (DSS), and LCR. Survival and LCR were calculated from the date of occurrence of the event or the date of the last follow-up. Tumor recurrence was defined as the emergence of further disease at least 6 months following complete TLM resection. For the LCR, local recurrence or presence of residual lesions was counted as an event. For the larynx preservation rate, only total laryngectomy was counted as an event. Each patient was followed until death or up to December 2021. Log-rank statistics were used to examine the relationship and determine statistical significance. A value of *p* < 0.05 was considered statistically significant.

Since selection bias is inevitable among retrospective studies. In order to improve the comparability between tumors treated with TLM or OPL, a stratified statistical analysis of items in the baseline data was performed to compare the differences between subcategorical variables and tumor outcomes between the two groups.

## Results

### Characteristics and treatment details of eligible patients

In the TLM group, a total of 55 patients matched the criteria for inclusion and exclusion. A total of 40 patients in the OPL group who met the inclusion and exclusion criteria and matched the clinical variables of TLM group, including age, gender, history of smoking and drinking, initial treatment modality, anterior commissure invaded situation and rT stage, were collected. And the final population included 92 males and 3 females. The average recurrent tumor duration was 20.69 ± 28.2 months (range, 6–144). The mean age at the time of diagnosis of recurrence was 61.11 ± 9.49 years (range, 39–81) in the TLM group, and 61.65 ± 8.24 years (range 41–75) in the OPL group. Detailed characteristics of the enrolled patients are shown in Table 1.

**Table 1:** Demographical and clinical data of the patients with recurrent glottic squamous cell cancer by treatment group

Variables	TLM (n = 55)	OPL (n = 40)	*P* value
Age of recurrence, y^†^			0.773
Mean ± SD	61.11 ± 9.49	61.65 ± 8.24	
Range	39–81	41–75	
Sex, male (%)^‡^	53 (96.4)	39 (97.5)	1.0
Smoking (%)^§^	32 (58.2)	23 (57.5)	0.947
Drinking (%)^§^	29 (52.7)	15 (37.5)	0.142
Initial treatment modality^‡^			0.679
RT alone	6 (10.9)	5 (12.5)	
TLM	47 (85.5)	35 (87.5)	
OPL	2 (3.6)	0 (0)	
rT stage of recurrence^‡^			0.082
rTis	2 (3.6)	0	
rT1a	20 (36.4)	10 (25)	
rT1b	25 (45.5)	16 (40)	
rT2	8 (14.5)	14 (35)	
Anterior commissure invaded of recurrence (%)^§^	24 (43.6)	20 (50)	0.677

Data are presented as number, mean ± standard deviation, or number (percentage)

*TLM* Transoral laser microsurgery, *OPL* Open partial laryngectomy, *RT* radiation therapy, *TNM* tumor-node-metastasis staging (American Joint Committee on Cancer, 8th edition)

^†^*P* value based on Student's t test

^‡^*P* values are based on Fisher’s exact probability

^§^*P* values are based on chi-square test

### Survival

**Fig. 2:**
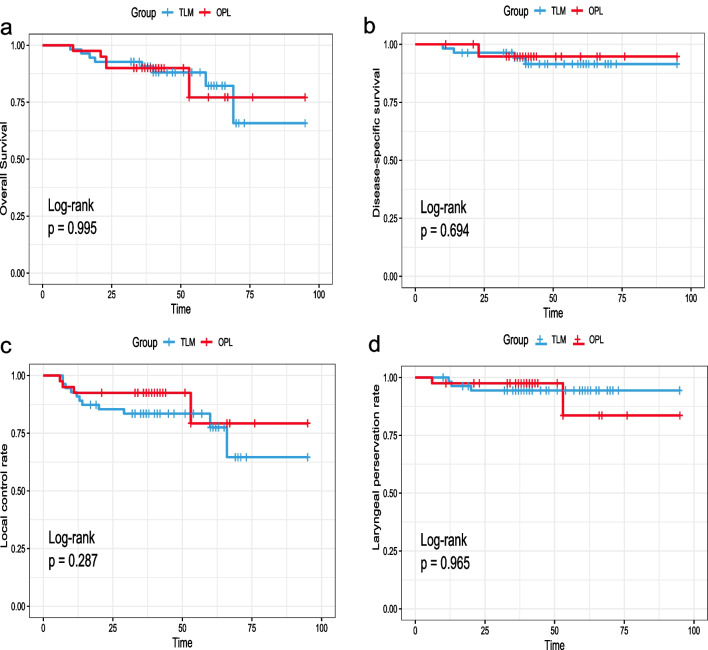
**a** Overall survival, **b** disease-specific survival, **c** local control rate and **d** laryngeal preservation rate for patients in the transoral laser microsurgery (TLM) or open partial laryngectomy (OPL) group

Following the surgical treatment of early local recurrent laryngeal cancer, either by TLM or by OPL, the patients were observed in a structured follow-up program for an average of 43.25 ± 15.17 months. During follow-up, 3 patient died due to pulmonary disease, myocardial infarction, or other causes in the TLM group and 5 patients died due to laryngeal cancer. In the OPL group, 2 patient died due to cerebral infarction or lung cancer, and 3 patients died due to laryngeal cancer. The 5-year OS and DSS were 65.8% and 91.5%, respectively, in the TLM group; and 77.1% and 94.7%, respectively, in the OPL group. There was no significant difference in the survival rate between the TLM subgroups and the OPL group (see Fig. 2, Table 2).

**Table 2:** Univariate analysis of the impact of different surgical methods on survival, local control, and larynx preservation outcome measures as assessed using log-rank tests

Parameter	Treatment group	Outcome (%)	*P* value
Overall survival	TLM (n = 55)	65.8	0.995
	OPL (n = 40)	77.1	
Disease-specific survival	TLM (n = 55)	91.5	0.694
	OPL (n = 40)	94.7	
Local control rate	TLM (n = 55)	64.6	0.287
	OPL (n = 40)	79.3	
Laryngeal preservation rate	TLM (n = 55)	94.4	0.965
	OPL (n = 40)	83.6	

*TLM* Transoral laser microsurgery, *OPL* Open partial laryngectomy

The *P* values for differences were tested with the Kaplan–Meier log-rank test

### Local control and larynx preservation

Following the first salvage TLM, 11 patients (20%) in the TLM group developed a second local recurrence, and the median time from the treatment of the first to the second recurrence was 11.64 months. After the first salvage OPL, 4 patients (10%) developed a second local recurrence, and the time to local relapse was 19.25 months. In the TLM and OPL groups, the local control rates after 5 years were 77.5% and 79.3%, respectively, with no significant differences (Table 2).

In the TLM group, 1 patient with secondary recurrence progressed to T_3_ stage, and there was no surgical indication for partial resection; therefore, total laryngectomy was performed. Similarly, 1 patient in the TLM group and 2 patients in the OPL group had secondary recurrence and progressed to rStage IV, requiring total laryngectomy. The laryngeal preservation rate (LPR) in the TLM and OPL groups was 94.4% and 83.6%, respectively, with no significant differences (Table 2).

To further improve the comparability between TLM and OPL and to reveal the reliability of salvage TLM, items including initial treatment modality, rT stage of recurrence, and anterior commissure invasion of recurrence were tested with stratified analysis. As shown in Table 3, the two groups of patients were grouped according to different characteristics, and comparative analysis was conducted. The results showed that there was no significant difference in tumor outcomes between the two groups in terms of individual items or their factors.

**Table 3:** Tumor outcomes in the two treatment groups according to different factors

Factor	*P* value			
	OS	DSS	LCR	LPR
*Initial treatment modality*				
RT alone (n = 11)	0.654	0.654	0.727	0.176
TLM (n = 82)	0.772	0.711	0.245	0.319
OPL (n = 2)	NA	NA	NA	NA
*rT stage of recurrence*				
rTis (n = 2)	NA	NA	NA	NA
rT1a (n = 30)	0.17	0.221	0.092	0.48
rT1b (n = 41)	0.944	0.816	0.998	0.861
rT2 (n = 22)	0.148	1	0.197	0.197
*Anterior commissure invaded of recurrence*				
Yes (n = 44)	0.995	0.739	0.946	0.687
No (n = 51)	0.819	0.93	0.293	0.859

*OS* Overall survival, *DSS* Disease-specific survival, *LCR* Local control rate, *LPR* Laryngeal preservation rate

The *P* values for differences were tested with the Kaplan–Meier log-rank test

### Anterior commissure involvement in patients with salvage TLM

24 of the 55 patients (43.64%) in the TLM group had anterior commissure involvement. However, this appears to have had no influence on the tumor outcome of salvage TLM. In the TLM group, the 5-year LCR of patients with or without anterior commissure involvement was 77.3% versus 61% (*p* = 0.361), the 5-year OS was 75.3% versus 84.5% (*p* = 0.898), and DSS was 78% versus 88.2% (*p* = 0.768), respectively. In addition, as shown in Table 3, there appeared to be no difference in tumor outcomes between the TLM and OPL groups of patients who had been anterior commissure involvement.

### Complications and hospitalization duration

In the TLM group, one patient (1.82%) developed glottis stenosis on the 5th month postoperatively and recovered well after transoral endoscopic microsurgery. A total of seven complications occurred in the OPL group, including two cases (5%) of postoperative laryngeal hemorrhage. One patient (2.5%) required treatment by an anesthesiologist owing to intolerable incision pain. Three patients (7.5%) had aspiration pneumonia due to postoperative aspiration. One patient developed submaxillary local inflammatory edema and one developed incision infection (2.5%). All patients were cured following analgesia, procoagulant, and antibiotic treatment. No patients died from bleeding, aspiration, and/or airway obstruction in either group. The TLM group showed better outcomes with a significantly lower complication rate, compared to those of the OPL group (*p* < 0.001).

The duration of hospitalization in the TLM group (5.42 ± 2.26 days) was significantly shorter than that in the OPL group (12.8 ± 4.12 days) (*p* < 0.001).

### Differences in HRQOL between TLM and OPL patients

The EORTC QLQ-HN35 scale was used to evaluate the HRQOL of the patients. A total of 53 and 35 patients in the TLM and OPL group, respectively, completed the scale successfully. Compared with the OPL group, the TLM group showed greater improvements in postsurgical HRQOL, especially in language, swallowing, social eating, and sense of pain (*p* = 0.022, 0.001, 0.001, and 0.024, respectively). As shown in Table 4, there were no significant differences in other domains or single-symptom items. Since none of the patients included in this study had a nasogastric tube or postsurgical analgesia, these two items were deleted from the list.

**Table 4:** Comparison of Domains in EORTC QLQ-H&N35 Affected by different surgical methods

Symptom scales/items	TLM (n = 53)		OPL (n = 35)		*P* value
	Mean	SD	Mean	SD	
Pain	3.46	8.40	7.86	11.78	0.024*
Swallowing	2.04	8.48	6.67	9.86	0.001*
Senses problems	5.35	12.13	14.29	21.82	0.051
Speech problems	22.64	18.1	32.7	20.51	0.022*
Trouble with social eating	1.89	5.07	7.86	7.27	0.001*
Trouble with social contact	3.4	6.62	4.38	6.85	0.418
Less sexuality	16.98	19.74	24.29	24.37	0.165
Teeth	11.32	17.23	17.14	27.26	0.552
Opening mouth	1.89	7.78	5.71	12.75	0.084
Dry mouth	15.72	22.27	13.33	20.13	0.634
Sticky saliva	11.95	21.77	12.38	22.99	0.974
Coughing	14.47	21.19	17.14	24.75	0.716
Felt ill	13.84	23.05	26.67	36.87	0.151
Nutritional supplements	3.77	19.24	11.43	32.28	0.166
Weight loss	9.43	29.51	14.29	35.5	0.485
Weight gain	16.98	37.91	11.43	32.28	0.475

*TLM* Transoral laser microsurgery, *OPL* Open partial laryngectomy

*These figures indicate statistical significance. The *P* values for differences were tested with the Mann Whitney U test

### Differences in VHI between patients treated with TLM and OPL

The scores in all domains of the VHI scale were compared to explore the differences in postsurgical QOV between patients with recurrent glottic cancer who had undergone TLM or OPL. Patients who had TLM had significantly lower functional, psychological, emotional, and total VHI scores compared with the OPL group, indicating that the QOV of the TLM group was significantly better than that of the OPL group (*p* < 0.001) (Table 5).

**Table 5:** Comparison of Domains in Voice Handicap Index Affected by different surgical methods

Voice Handicap Index	TLM(n = 53)		OPL(n = 35)		*P* value
	Mean	SD	Mean	SD	
Functional	9.83	5.08	14.86	5.49	< 0.001*
Physical	10.19	5.14	14.46	6.23	0.002*
Emotional	7.85	3.86	11.37	4.25	< 0.001*
Total scores	27.87	11.26	40.69	14.45	< 0.001*

*TLM* Transoral laser microsurgery, *OPL* Open partial laryngectomy

*These figures indicate statistical significance. The *P* values for differences were tested with the Mann Whitney U test

## Discussion

A retrospective analysis was used in this study to compare the therapeutic effects of salvage TLM and OPL. The results showed that for early local recurrence of GSCC, there were no significant differences between the TLM and OPL groups in terms of survival, local control, or laryngeal preservation. Furthermore, the stratified analyses showed that the TLM group’s results were similar to those of the OPL group, which further validated the results of the TLM.

Recent studies have shown that the 5-year OS and DSS rate following salvage TLM are 64.8–89.9% and 79.6–97.9%, respectively [25, 26]. Similar to the previously reported results, patients with early local recurrence of GSCC after initial TLM had good postoperative results with salvage TLM in this study. Zhong et al. [27] included 10 studies in their meta-analysis and reported that salvage TLM showed good results for local control, laryngeal preservation, and survival, and that TLM is a potentially effective option for early recurrent laryngeal cancer after initial treatment (regardless of the treatment method), which was consistent with our findings.

However, some studies that have drawn different conclusions warrant mention. Motamed et al. [28] pointed out that one disadvantage of salvage TLM is the high local recurrence rate, suggesting that local control rates of total laryngectomy are superior to laryngectomy. A systematic review and meta-analysis by Ramakrishnan et al. [29] showed a downward trend in LCR of TLM compared to that of OPL. Compared to the results of previous reports, our study appears to have achieved better oncologic outcomes, which could be due to the strict surgical indications developed for salvage TLM and the fact that all salvage TLM was performed by two experienced and skilled specialists to ensure surgical quality. Additionally, a strict and standardized postoperative follow-up after laryngeal cancer surgery and timely observation of tumor progression may have led to the good tumor outcome. Although retrospective studies are biased in nature, by stratified comparison the items in the baseline data of the TLM group and the OPL group, we had reason to believe that salvage TLM is an effective surgical treatment, especially for early local recurrence of laryngeal cancer. Particularly important is that based on stratified analysis, we found that anterior commissure involvement does not appear to affect the outcome of salvage TLM. However, it is worth mentioning that in our center, the initial treatment of early laryngeal cancer is more inclined to surgery. Only 6 patients in the TLM group were treated with radical radiotherapy as primary treatment (no outcome event occurred during follow-up). Given that many centers around the world use radiation therapy as the primary treatment for early stage laryngeal cancer, the use of salvage TLM in patients with early recurrent laryngeal cancer who received radiotherapy needs to be further investigated.

At present, there are few studies examining salvage TLM for laryngeal cancer, and the clinical norms, guidelines and techniques are not mature. There is still a lack of guidance on when to use salvage TLM and how to select suitable patients. In addition, the high requirements for minimally invasive surgical techniques have greatly limited the application of minimally invasive rescue in clinical practice. For example, the surgeon should be a senior surgeon with extensive experience in microsurgery. Surgeons are required to be able to fully expose the tumor through direct laryngoscope, master laser surgery technology, and be able to accurately judge the scope and depth of direct laryngoscope resection combined with imaging data. It should be noted that the positive margin rate of salvage surgery can be as high as 40%. Although the surgical resection margins were carefully ensured during the surgery and were assisted with frozen biopsy, there was still a failure in obtaining safe margins in 22% of the patients, as observed microscopically [30, 31]. Therefore, open surgery is preferred for advanced recurrent laryngeal cancer due to the inability of TLM to solve problems such as surgical field exposure, complete tumor resection, and safe resection margins. After analysis of the treatment points and key issues of salvage TLM, we proposed the surgical indications of salvage TLM and standardized the pre-treatment evaluation process and surgical techniques of salvage TLM for recurrent laryngeal cancer. After a full, comprehensive and careful evaluation of the characteristics of recurrent laryngeal cancer, we found that for early local recurrence of GSCC (rTis-2N0M0), standard salvage TLM can retain enough safe margins and completely remove the tumor. Compared with open surgery, there were no significant differences in LCR, OS, DSS and LPR in patients with early local GSCC (rTis-2N0M0) treated with salvage TLM.

According to our results, patients with salvage TLM had fewer complications, shorter hospital stays, and significantly improved survival and voice quality. After successful resection of laryngeal cancer, patients may experience severe dysfunction and/or changes in appearance. Therefore, the consideration of HRQOL and QOV is particularly important in evaluating the effect of laryngeal cancer treatment [32]. The QOL of patients following TLM is considered better than that following partial laryngectomy. However, few previous reports have assessed the impact of different salvage treatment methods on the QOL and QOV of patients with laryngeal cancer. Herein, the EORTC QLQ-HN&35 and the VHI-30 scale were used to evaluate the QOL and QOV of patients undergoing TLM and OPL. TLM showed greater improvements in QOL compared to OPL, especially in language, swallowing, social eating, and disease feeling. The QOV was also significantly better than that of the OPL group, and the VHI function, psychological, emotional, and total scores were significantly reduced (*p* < 0.001). In addition to reducing the duration of hospitalization and incidence of complications, salvage TLM is also beneficial in improving the QOL, which is mainly due to its minimally invasive characteristics.

This study has certain limitations. First, owing to the strict inclusion and exclusion criteria, the sample size of this study was small. Second, due to the retrospective nature of the analysis, several types of bias could not be avoided. Nevertheless, a clear trend consistent with previous reports was observed, which may clearly aid in the management of early local recurrence of glottic cancer using salvage TLM. We intend to increase the sample size in the future and design a prospective study to improve the relevant conclusions.

Notably, early local recurrence (rT_is-2_N_0_M_0_) of glottic squamous cell cancer after treatment is an indication for salvage TLM. If the recurrence site is extensive or has distant metastases, the patient may not be eligible for a minimally invasive procedure. Therefore, a strict and standardized postoperative follow-up after laryngeal cancer surgery is important. Early detection of recurrent or residual lesions can significantly improve the feasibility and success rate of salvage TLM, and clinicians should pay sufficient attention to this. We recommend that patients who undergo salvage TLM should meet the following conditions: (1) early local recurrence (rT_1_-2N_0_M_0_) of glottic cancer after initial treatment; (2) no involvement of the laryngeal cartilage scaffold, pre-epiglottic space, or paraglottic space, and no distant metastasis; and (3) a tumor that can be fully exposed and completely excised under direct laryngoscopy, according to the preoperative assessment.

## Conclusions

Compared to the outcomes of OPL, salvage TLM has fewer serious complications, a shorter hospitalization duration, and significantly better functional results for vocalization and swallowing. It can be used as an effective choice for the treatment of early local recurrence of glottic squamous cell cancer. Early detection and accurate assessment are the key to improving the success rate of salvage TLM.

## Supplementary Information

**Additional file 1: Video P1.** Salvage transoral laser microsurgery for early local recurrence of laryngeal cancer.

**Additional file 2: Video P2.** Salvage transoral laser microsurgery for early local recurrence of laryngeal cancer.

## Data Availability

The datasets used and/or analysed during the current study are available from the corresponding author on reasonable request.

## Abbreviations

EORTC QLQ-H&N35: European Organization for Research and Treatment of Cancer Quality of Life Questionnaire-Head and Neck
DSS: Disease-specific survival
GSCC: Glottic squamous cell cancer
HRQOL: Health-related quality of life
LCR: Local control rate
NBI: Narrow-band imaging
OPL: Open partial laryngectomy
OS: Overall survival
QOL: Quality of life
LPR: Laryngeal preservation rate
QOV: Quality of voice
TLM: Transoral laser microsurgery
VHI: Voice handicap index
